# Diffusion lacunae: a novel MR imaging finding on diffusion-weighted imaging for diagnosing placenta accreta spectrum

**DOI:** 10.1007/s11604-024-01657-6

**Published:** 2024-09-11

**Authors:** Yuko Iraha, Shinya Fujii, Nanae Tsuchiya, Kimei Azama, Eri Yonamine, Keiko Mekaru, Tadatsugu Kinjo, Masayuki Sekine, Akihiro Nishie

**Affiliations:** 1 Department of Radiology, Graduate School of Medical Science, University of the Ryukyus, 207 Uehara, Nishihara, Okinawa Japan; 2 Division of Radiology, Department of Multidisciplinary Internal Medicine, Faculty of Medicine, Tottori University, 36-1, Nishi-Cho, Yonago, Tottori Japan; 3 Department of Obstetrics and Gynecology, Graduate School of Medical Science, University of the Ryukyus, 207 Uehara, Nishihara, Okinawa Japan

**Keywords:** Magnetic resonance imaging, Diffusion-weighted imaging, Placenta accreta spectrum, Placental lacunae, Flow voids

## Abstract

**Objective:**

To evaluate the usefulness of novel diffusion-weighted imaging (DWI) findings for diagnosing placenta accreta spectrum (PAS).

**Materials and methods:**

This retrospective study included 49 pregnant women with suspected PAS who underwent 1.5 T placental MRI. Diffusion lacunae were defined as intraplacental areas showing hypointensity on DWI and hyperintensity on the apparent diffusion coefficient map. Two radiologists evaluated the number and size of placental lacunae on DWI, and flow void in the diffusion lacunae on T2-weighted imaging. The radiologists also evaluated established MRI features of PAS described in the SAR-ESUR consensus statement. Pearson's chi-square test or Mann–Whitney U test was used to compare findings between patients with and without PAS. Interobserver reliability for DWI and established MRI features was also assessed. Optimal thresholds for the number and maximum size of diffusion lacunae for differentiating PAS from the no-PAS group were determined using receiver operating characteristic curve analyses.

**Results:**

Eighteen patients were diagnosed with PAS, and 31 patients with placental previa without PAS. The number and maximum size of diffusion lacunae were significantly larger in patients with than in patients without PAS (p < 0.0001). Combining assessment of the number of diffusion lacunae with assessment of their maximum size yielded a diagnostic performance with sensitivity, specificity and accuracy of 83%, 94% and 90%, respectively. Flow voids within the diffusion lacunae had sensitivity, specificity and accuracy of 88%, 84% and 86%, respectively.

**Conclusion:**

The number and size of diffusion lacunae, and T2 flow void in diffusion lacunae may be useful findings for diagnosing PAS.

## Introduction

Placenta accreta spectrum (PAS) is a spectrum of conditions characterized by abnormal implantation of chorionic villi on the myometrium without an intervening decidua basalis. As is widely known, the main risk factors for PAS are placenta previa and prior cesarean delivery. The risk of PAS is strongly associated with the number of previous cesarian section [1]. Additionally, the incidence of PAS has increased ten-fold in the last 50 years due to a rapidly increasing rate of cesarean delivery [2–5], with the incidence 1 in 4017 women in the 1970s to 1 in 313 between 2015 to 2017 [6]. Thus, the incidence of PAS is expected to continue increasing. PAS is associated with 18- fold increase in maternal morbidity and with up to 30% mortality rate, particularly when prenatal diagnosis is missed [7]. Accurate prenatal diagnosis of PAS is crucial for the management including comprehensive multidisciplinary approach such as cesarean hysterectomy [8–10].

Ultrasound (US) is the first-choice imaging modality for PAS. Placental lacunae are the most common US sign in the diagnosis of PAS [11]. The lacunae are thought to represent enlargement of intervillous spaces and appear as intraplacental sonolucent areas on US. In invasive PAS, the placenta reaches deep into the myometrium. Consequently, the maternal blood flows directly into the intervillous space at a higher velocity from the radial arteries rather than the spiral arteries, which distorts the interlobar septa and leads to placental lacunae [11]. There are usually multiple placental lacunae, with variations in the total number, shape, location, and the degree of blood flow. Presence of numerous, large and irregular lacunae, and turbulent flows within lacunae are associated with invasive placentation [11–15].

Magnetic resonance (MR) examination may compliment US and provide useful information for surgical planning of PAS [16–22]. Recently, the Society of Abdominal Radiology (SAR) and European Society of Urogenital Radiology (ESUR) established a consensus statement and proposed seven MR imaging findings such as placental bulge and T2-dark bands as recommended signs for the diagnosis of PAS [23]. These findings are usually evaluated using T2-weighted imaging (T2WI), due to its excellent contrast resolution. Meanwhile, diffusion-weighted imaging (DWI) is considered a highly promising sequence in gynecological imaging [24]. However, few reports have investigated its clinical utility of DWI in diagnosis of PAS [25, 26]. In our recent evaluations of patients suspicious for PAS, we have often noticed placental lacunar-like structures with hypointense ovoid or irregular spaces on DWI and flow void on T2WI within the DWI hypointense area, which may correspond to US lacunae and lacunar blood flow, respectively. Thus, we define the hypointense area on DWI as “diffusion lacunae”. In our present investigation, we evaluated the usefulness of diffusion lacunae and T2 flow void within diffusion lacunae for the diagnosis of PAS.

## Materials and methods

### Patients

**Fig. 1:**
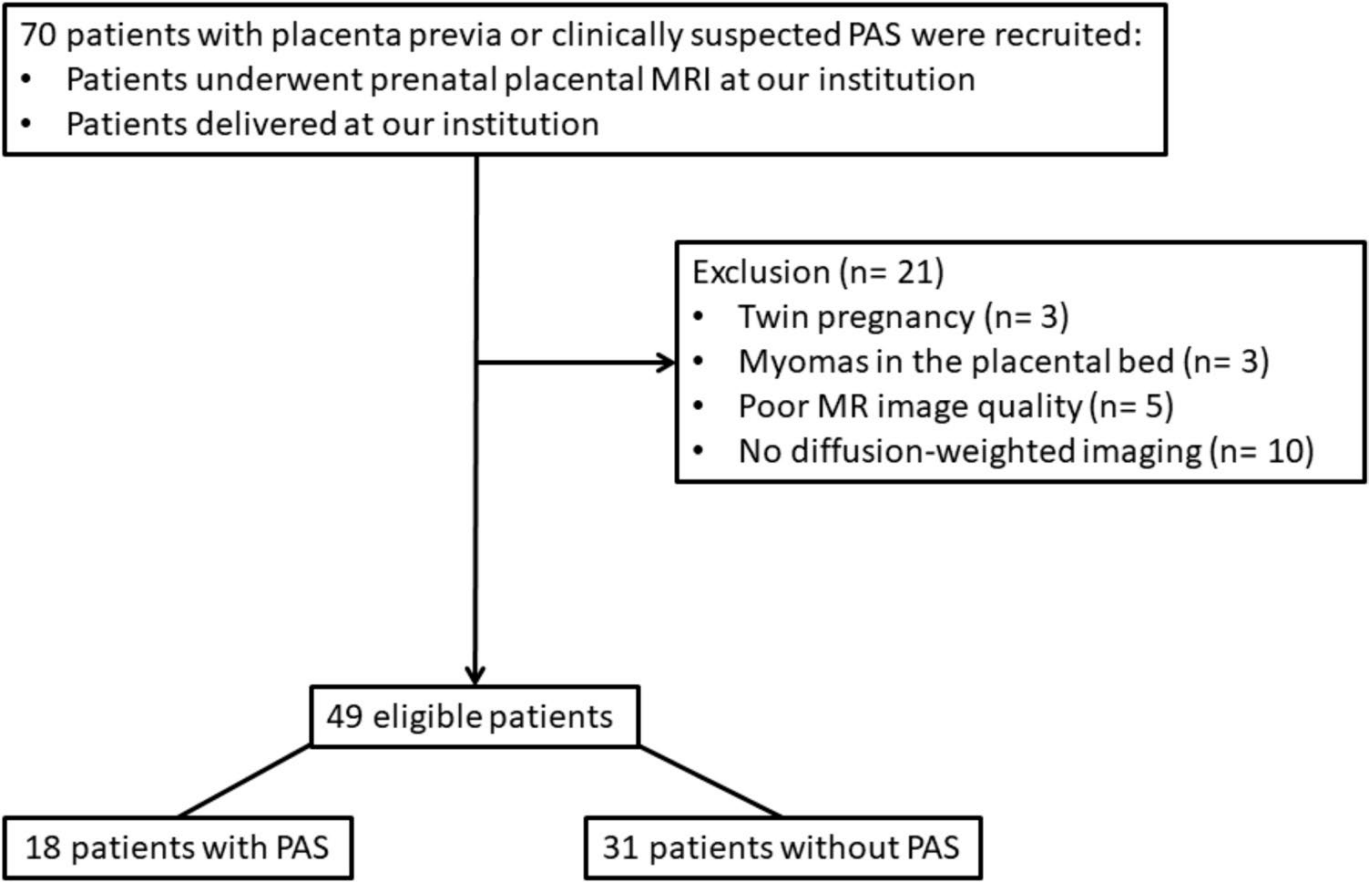
Flowchart of inclusion and exclusion criteria

This study was approved by our institutional review board. Written informed consent was waived due to the retrospective nature of this study. We initially enrolled 70 consecutive patients with placenta previa or clinically suspected of PAS, who underwent prenatal placental MR imaging and delivered at our institution between January 2011 and December 2022. After excluding patients with twin pregnancy (n = 3), myomas in the placental bed (n = 3), or poor MR image quality (n = 5), and patients who did not obtain DWI (n = 10), a study group of 49 patients was analyzed in this study. Figure 1 provides a patient flow chart with inclusion criteria and exclusions.

We also recorded the following clinical data: maternal age, gravidity, parity, number of previous cesarean deliveries, previous uterine surgery, pregnancy by assisted reproductive technology, presence of a low-lying placenta or placenta previa, main placental location of previa, gestational age at MR imaging, gestational age at delivery, types of delivery and intraoperative blood loss.

### MR imaging protocols

All MRI examinations were acquired at a 1.5 T unit (Magnetom Avanto, Siemens Healthcare, Erlangen, Germany) with body array coils. The following sequences were included: axial, coronal and sagittal T2-weighted half-Fourier acquisition single-shot turbo spin echo (HASTE) and true fast imaging in steady-state precession (True-FISP), sagittal and/or axial and/or coronal T1-weighted gradient-echo and axial DWI. The parameters for HASTE, True-FISP, and DWI are shown in Table 1. DWI was obtained with b-values of 0, 800 and 1000 s/mm^2^ in 40 patients and b-values of 0 and 1000 s/mm^2^ in 9 patients.

**Table 1:** MR imaging parameters

Sequence	HASTE	True-FISP	DWI
TR/TE (ms)	1800/66–69	3.4–3.6/1.69–1.82	8000/75–90
FA (degree)	150	70	90
FOV (mm)	420–498 × 320–380	420–498 × 320–380	380–498 × 280–380
Matrix	208–256 × 256	208–256 × 256	104–128 × 128
NEX	1	2	2
Slice thickness (mm)	4–8	4–8	4–8
b factor			0, 800, 1000

*HASTE*, T2-weighted half-Fourier acquisition single-shot turbo spin echo; *True-FISP*, true fast imaging in steady-state precession; *DWI*, diffusion weighted imaging; *TR/TE*, repetition time/echo time; *FA*, frip angle; *FOV*, field of view; *NEX*, number of excitations

### Imaging analysis

Imaging analysis was carried out independently by two experienced radiologists (with 10 and 18 years of reader experience in body MR imaging, respectively) who were blinded to the clinical data of patients.

**Fig. 2:**
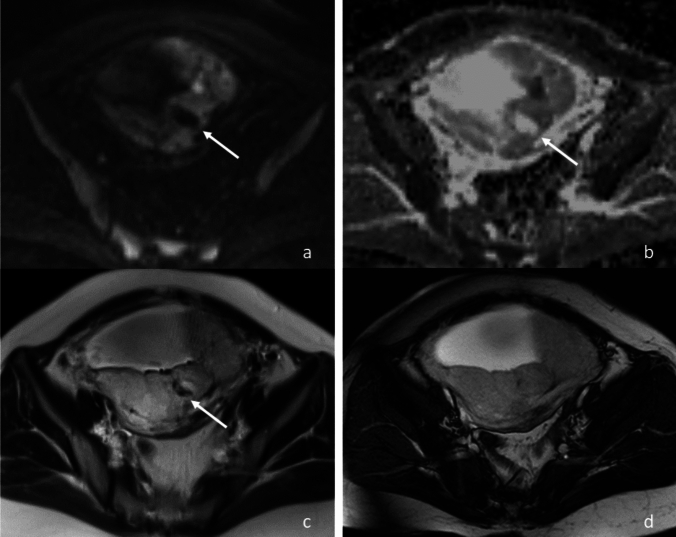
A 30-year-old woman with placenta previa and placenta percreta. **a** Axial diffusion-weighted image shows a hypointense ovoid space (arrow) in the placenta. **b** The apparent diffusion coefficient map shows hyperintense space (arrow) corresponding to the hypointense diffusion area in (**a**), indicating diffusion lacunae. **c** Axial T2-weighted half-Fourier acquisition single-shot turbo spin echo shows flow void in the diffusion lacuna (arrow). **d** True fast imaging in steady-state precession shows high signal intensity throughout the placenta, including the diffusion lacuna

**Fig. 3:**
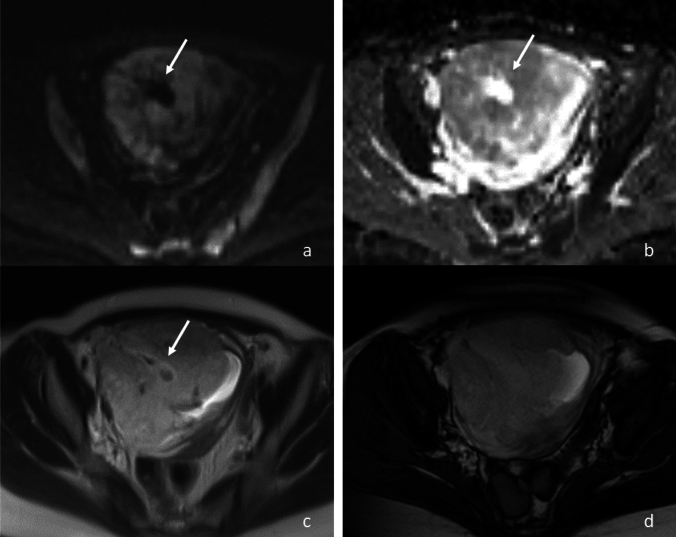
A 39-year-old woman with placenta previa and placenta accreta. **a** Axial diffusion-weighted image shows a hypointense area (arrow) in the placenta. **b** The apparent diffusion coefficient map shows hyperintense space (arrow) corresponding to the hypointense diffusion area in (**a**), indicating diffusion lacuna. **c** Axial T2-weighted half-Fourier acquisition single-shot turbo spin echo shows faint flow void in the diffusion lacuna (arrow). **d** True fast imaging in steady-state precession shows high signal intensity throughout the placenta, including the diffusion lacuna

We defined the following findings as diffusion lacunae: intraplacental ovoid or irregular hypointense spaces equal to amniotic fluid on DWI with high b values, which appear as hyperintensities on the ADC map and True-FISP (Fig. 2, 3). The spaces with hypointensity on both DWI and True-FISP were excluded from the assessment due to possible T2-dark bands. The spaces with less than 1 cm in maximum diameter, or with unclear boundaries were excluded from the assessment because the size of placental lacunae on US is defined as ≥ 1 cm in diameter [27]. Additionally, the spaces that were mainly distributed peripherally and not surrounded by placental tissue were excluded from the assessment in order to exclude placental marginal sinus. The spaces with a tubular configuration were also excluded from the assessment to exclude intraplacental vessels. Two readers independently evaluated the number of the diffusion lacunae and the largest maximum diameter (“size” described as below) among all diffusion lacunae. In case of large diffusion lacunae extending over two slices on axial DWI, we used sagittal and coronal HASTE as reference to measure the maximum diameter. Namely, the lacunae were projected and measured on coronal and sagittal plains when the maximum diameter was in the left–right and antero-posterior directions, respectively. Then the mean values between them were calculated.

Subsequently, two radiologists independently evaluated flow void within the diffusion lacunae on HASTE and True-FISP, with reference to DWI and the ADC map. The flow void showed hypointensity on HASTE and hyperintensity on True-FISP (Fig. 3). When the evaluations of the two readers differed, agreement was reached by consensus.

We also evaluated several MR features that were previously reported to be associated with PAS, and which were highly recommended as standard MR imaging findings in the joint consensus statement of SAR-ESUR [23]. These included T2-dark bands, placental bulge, loss of T2 hypointense interface, myometrial thinning, bladder wall interruption, focal exophytic mass, and abnormal vascularization of the placental bed. Additionally, abnormal intraplacental vascularity was evaluated because this finding may be associated with flow void within the lacunae [16], although the finding was categorized as uncertain in the SAR-ESUR consensus statement. Two readers independently evaluated these MR features. When their evaluations diverged, the two readers reached agreement by consensus. The definitions of all the MR features are shown in Table 2 based on the consensus statement [23].

**Table 2:** Definition of standard MR imaging findings (23)

MR feature	Definition
T2-dark bands	One or more areas of hypointensity on T2WI, which are usually linear in configuration and often contact the maternal surface of the placenta
Placental bulge	Deviation of the uterine serosa from the expected plane caused by an abnormal bulge of placental tissue toward adjacent organs, typically toward the bladder and parametrium. The uterine serosa may be intact, but the outline shape is distorted
Loss of T2 hypointense interface	Loss of a thin dark line behind the placental bed, as seen on T2WI, or loss of clear boundaries between placenta and uterine myometrium on T2WI
Myometrial thinning	Thinning of the myometrium over the placenta to less than 1 mm or even invisible
Bladder wall interruption	Irregularity or disruption of the normal hypointense bladder wall, which can be accompanied by blood products in the bladder lumen
Focal exophytic mass	Placental tissue seen protruding through the uterine wall and extending beyond it Most commonly seen inside at least partially filled urinary bladder and laterally into the parametrium
Abnormal vascularization of the placental bed	Prominent vessels in the placental bed with disruption of the uteroplacental interface. They may extend to the underlying myometrium to a variable degree, reaching up to the uterine serosa; and may be accompanied by extensive neovascularization around the bladder, uterus, and vagina
Abnormal intraplacental vascularity	Abnormal vessels, tortuous enlarged flow voids on T2WI deep within the placenta, more than 6 mm in diameter

*T2WI*, T2-weighted images

### Standard of reference for PAS

We used operative and pathological findings as the gold standard diagnostic data according to the International Federation of Gynecology and Obstetrics (FIGO) classification [28]. We reviewed the presence and depth (including placenta accreta, increta, and percreta) of PAS on the operative and pathologic reports.

### Statistical analysis

Statistical analysis was performed using JMP 17. software (SAS Institute Inc.). A value of *P* < 0.05 was considered to be statistically significant. Categorical variables were evaluated using the Pearson χ2 test or Fisher’s exact test. Interobserver agreement in categorical variables was assessed by using kappa statistics.

Continuous variables including the number and size of the diffusion lacunae were assessed using Student’s t-test or Mann–Whitney U test. Intraclass correlation coefficients (ICCs) were used for assessing these agreements.

To evaluate the diagnostic utility of the number and size of diffusion lacunae, receiver operating characteristic (ROC) curve analyses and calculation of the area under the curve (AUC) were performed. We determined the optimal thresholds for differentiating PAS from the no-PAS group by maximizing the Youden index.

The PAS-diagnostic performance of the novel MR imaging findings related to diffusion lacunae and the standard MR imaging findings shown in Table 2 was assessed according to the sensitivity, specificity, positive predictive value, negative predictive value, and accuracy.

## Results

Eighteen patients were diagnosed with PAS based on the operative and pathological findings. Meanwhile, 31 patients underwent cesarean section with placental previa and served as the no-PAS group. Among the 18 patients diagnosed with PAS, 16 patients underwent cesarean hysterectomy, including pathologically diagnosed with placenta accreta (n = 4), placenta increta (n = 5), and placenta percreta (n = 7). Two patients were clinically diagnosed with placenta accreta since their placenta could not be fully removed during cesarean section; one of these patients required uterine artery embolization and the other required uterine balloon tamponade due to heavy bleeding after manual removal of the placenta. The patient characteristics were shown in Table 3.

**Table 3:** Patient demographic and clinical characteristics

	PAS (n = 18)	No PAS (n = 31)	*P* value
Age*	35.2 ± 4.3 (28–41)	34.5 ± 4.0 (27–41)	0.569
Gravidity†	4.2 (1–10)	1.8 (0–4)	< 0.0001
Parity†	2.5 (0–7)	0.9 (0–4)	0.0001
Prior cesarean section†	1.8 (0–4)	0.1 (0–1)	< 0.0001
Other prior uterine surgery or procedure	7	10	0.758
ART pregnancy	3	6	
Placenta previa	17	31	
Low-lying placenta	1	0	
Main placental location of previa			0.0121
Anterior	11	7	
Posterior	5	20	
Anterior-lateral	1	4	
Gestational age at MRI (weeks)†	31 (28–34)	32 (27–37)	0.0135
Gestational age at delivery (weeks)†	34 (32–38)	37 (33–38)	< 0.0001
Types of delivery			< 0.0001
Cesarean section	2	31	
Cesarean hysterectomy	16	0	
Depth of placental invasion			
Accreta	6	-	
Increta	5	–	
Percreta	7	–	
Blood loss during delivery* (ml)	5238 ± 3290 (1300–11800)	1706 ± 841 (540–4060)	< 0.0001

*ART*, assisted reproductive technology; *PAS*, placenta accreta spectrum

^*^ Values are expressed as means ± standard deviations, with ranges in parentheses

^†^Values are means, with ranges in parentheses

﻿Inter-observer agreement was substantial or almost perfect for the evaluation of most MRI features suggesting PAS except loss of T2 hypointense interface and abnormal intraplacental vascularity (kappa coefficient: 0.55–0.86). The ICCs in the number and size of diffusion lacunae between two radiologists were almost perfect and substantial (0.83 and 0.64), respectively (Table 4).

**Table 4:** Interobserver reliability for MR imaging findings

MR imaging findings	κ/ ICC value	
Novel MR imaging findings		
Number of the diffusion lacunae*	0.83	
Size of the diffusion lacunae*	0.64	
Flow voids within the diffusion lacunae	0.61	
Standard MR imaging findings		
T2-dark bands	0.83	
Placental bulge	0.86	
Loss of T2 hypointense interface	0.58	
Myometrial thinning	0.8	
Bladder wall interruption	0.79	
Focal exophytic mass	0.61	
Abnormal vascularization of the placental bed	0.81	
Abnormal intraplacental vascularity	0.55	

*ICC*, intraclass correlation coefficients. *Assessed by ICC

The kappa values and ICC were interpreted as follows:

0.00–0.20, slight agreement; 0.21–0.40, fair agreement; 0.41–0.60, moderate agreement; 0.61–0.80, substantial agreement; 0.81 or more, almost perfect agreement

**Fig. 4:**
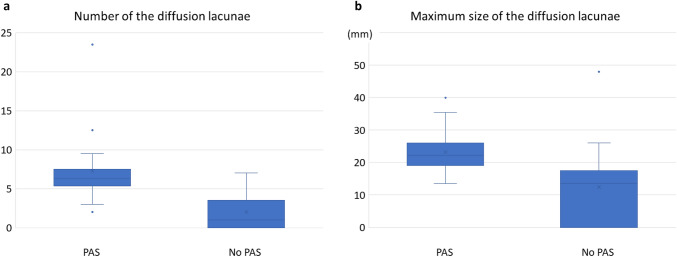
Box and whisker plots of number and maximum size of the diffusion lacunae. **a** The mean numbers of diffusion lacunae in the PAS and no-PAS groups were 7.3 and 2, respectively. The number of diffusion lacunae was significantly higher in the patients with PAS (p < 0.0001). **b** The mean maximum sizes of diffusion lacunae in the PAS and no-PAS groups were 24 mm and 12 mm, respectively. The maximum size of diffusion lacunae was significantly larger in the patients with PAS (p < 0.0001)

The mean number and size of the diffusion lacunae in the PAS group were 7.3 and 24 mm, respectively. Meanwhile, the mean number and size in the no-PAS group were 2 and 12 mm, respectively. The number and size of the diffusion lacunae were significantly larger in the patients with PAS than in the patients without PAS (p < 0.0001) (Figs. 4a and 4b). The AUC using ROC analysis for the prediction of PAS based on the number of the diffusion lacunae was 0.90, and the derived optimal cut-off value was 5. In addition, the AUC for prediction of PAS based on the size of the diffusion lacunae was 0.85, and the derived optimal cut-off value was 17 mm. Based on these cutoff values, we defined the presence of 5 or more diffusion lacunae and the presence of diffusion lacunae with 17 mm or greater size as predictive findings for PAS.

The rates of occurrence of all MR features are shown in Table 5. Flow void within diffusion lacunae was significantly more often observed in patients with PAS (Table 5). Significant differences were found in other novel and standard MR imaging findings between patients with PAS and the no-PAS group, except for bladder wall interruption (Table 5).

**Table 5:** Incidence of MR features

MR feature	PAS (n = 18)	No PAS (n = 31)	*P* value	
Novel MR imaging findings				
Number of diffusion lacunae ≥ 5	15	4	< 0.0001	
Size of diffusion lacunae ≥ 17 mm	16	8	< 0.0001	
Flow voids within diffusion lacunae	16	5	< 0.0001	
Combination of number and size*	15	2	< 0.0001	
Either number and size or flow void**	18	5	< 0.0001	
Combination of number and size and flow void***	13	2	< 0.0001	
Standard MR imaging findings				
T2-dark bands	16	5	< 0.0001	
Placental bulge	16	1	< 0.0001	
Loss of T2 hypointense interface	16	4	< 0.0001	
Myometrial thinning	18	23	< 0.0001	
Bladder wall interruption	2	0	0.0581	
Focal exophytic mass	8	0	< 0.0001	
Abnormal vascularization of the placental bed	13	2	< 0.0001	
abnormal intraplacental vascularity	7	2	< 0.0001	

*PAS*, Placenta accreta spectrum

^*^Combination of the number and size (both ≥ 5 and ≥ 17 mm) of diffusion lacunae

^**^Either combination of the number and size (both ≥ 5 and ≥ 17 mm) or flow void within diffusion lacunae

^***^Combination of the number and size (both ≥ 5 and ≥ 17 mm) and flow void within diffusion lacunae

The diffusion lacunae ≥ 5 in number and the lacunae ≥ 17 mm in size had a diagnostic performance with sensitivity of 83% and 89%, specificity of 87% and 74%, and accuracy of 86% and 80%, respectively. The combination of both parameters (≥ 5 in number plus ≥ 17 mm in size) yielded a diagnostic performance with a sensitivity of 83%, specificity of 94% and accuracy of 90% (Table 6). For the evaluation of flow voids within the diffusion lacunae, the sensitivity, specificity and accuracy were 88%, 84% and 86%, respectively (Table 6). In addition, either the combination of diffusion lacunae findings (≥ 5 in number plus ≥ 17 mm in size) or the flow voids yielded a sensitivity of 100%, a specificity of 84% and an accuracy of 90% (Table 6).

**Table 6:** Diagnostic performances of MR imaging findings

MR feature	Sensitivity	Specificity	PPV	NPV	Accuracy
Novel MR imaging findings					
Number of diffusion lacunae ≥ 5	83%	87%	79%	90%	86%
Size of diffusion lacunae ≥ 17 mm	89%	74%	67%	92%	80%
Flow voids within diffusion lacunae	88%	84%	76%	93%	86%
Combination of number and size*	83%	94%	88%	91%	90%
Either number and size or flow void**	100%	84%	78%	100%	90%
Combination of number and size and flow void***	72%	94%	87%	85%	86%
Standard MR imaging findings					
T2-dark bands	89%	84%	76%	93%	86%
Placental bulge	89%	97%	94%	94%	94%
Loss of T2 hypointense interface	88%	87%	80%	93%	88%
Myometrial thinning	100%	74%	69%	100%	84%
Bladder wall interruption	11%	100%	100%	66%	67%
Focal exophytic mass	56%	100%	100%	79%	84%
Abnormal vascularization of the placental bed	72%	94%	87%	85%	86%
abnormal intraplacental vascularity	39%	94%	78%	73%	73%

*NPV*, negative predictive value; *PPV*, positive predictive value

^*^Combination of the number and size (both ≥ 5 and ≥ 17 mm) of diffusion lacunae

^**^Either combination of the number and size (both ≥ 5 and ≥ 17 mm) or flow void within diffusion lacunae

^***^Combination of the number and size (both ≥ 5 and ≥ 17 mm) and flow void within diffusion lacunae

Regarding the standard MR imaging findings, placental bulge had the highest accuracy of 94%, followed by loss of T2 hypointense interface with 88%, abnormal vascularization of the placental bed with 86%, and T2-dark bands with 86%. The details of the diagnostic performance of standard MR imaging findings are shown in Table 6.

## Discussion

Our study revealed that the number and size of diffusion lacunae were significantly greater in patients with than those without PAS. Additionally, flow voids in the diffusion lacunae were significantly more often observed in PAS. Moreover, the combination of number and size of the diffusion lacunae (both ≥ 5 and ≥ 17 mm) yielded relatively high specificity and the second highest accuracy among all MR imaging findings. In addition, either the combination of diffusion lacunae findings or the flow voids yielded the highest sensitivity and the second highest accuracy among all MR imaging findings. Therefore, we consider that our novel MR imaging findings of the diffusion lacunae may be useful for the diagnosis of PAS.

Placental lacunae are the most common and key US sign in patients with PAS [29, 30]. We speculate that the diffusion lacunae observed in our study correspond to placental lacunae on US. Only DWI enables to distinguish the placental lacunae from villous tissue on MRI, whereas T2-weighted imaging does not. In normal placentation, the remodeled spiral arteries forming a low-resistance vascular network supply a high flow volume of maternal blood to the placenta without excessive velocity. Meanwhile, in invasive PAS, increased blood flow disrupts the normal lobular structure of the placenta, leading to enlarged intervillous spaces, as mentioned in introduction [11]. Thus, the lacunae are strongly related to invasive PAS [29, 30]. Consequently, the recognition of the lacunae is very important for the diagnosis as well as the understanding of the pathophysiology of PAS.

Combined evaluation both size and number of diffusion lacunae is considered important to distinguish placental lacunae from placental lakes. Placental lakes, which often observed in normal placentas, are considered to be intervillous vascular spaces as a result of intervillous circulatory dysfunction [31–33]. Both placental lakes and lacunae demonstrate sonolucent areas on US. Moreover, placental lakes and lacunae can coexist in the same placenta [32]. Therefore, it may be difficult to distinguish them even using MRI. However, larger lakes are often found mainly in areas of lower villous density under the fetal plate or in the marginal areas and usually not present in large numbers in a normal placenta [11, 19, 34]. In fact, the specificity and accuracy were increased by combined evaluation of the size and number of diffusion lacunae in our study.

Flow voids within diffusion lacunae were observed significantly more often in patients with PAS, suggesting that the presence of blood flow within diffusion lacunae may be a useful diagnostic finding. Many studies about PAS have shown that blood flow exists within the lacunae on US [11, 35]. Regarding MRI, Derman et al. [16] and Clark et al. [36] reported that the deeply located intraplacental signal voids on T2 HASTE indicate the blood flow within the placental lacunae. Lacunar blood flow is various from low-velocity flow to turbulent high-velocity flow [15, 37]. Furthermore, some reports have shown that the lacunae in invasive PAS often contain turbulent flow [38–40], which is in keeping with our results that flow voids within the diffusion lacunae were observed in most patients with PAS. In contrast, the blood flow observed in placental lakes consistently shows laminar low-velocity flow [31, 32], unlike that observed in placental lacunae. In actuality, flow voids are rarely observed in patients without PAS. Therefore, combined assessment of the number and size of diffusion lacunae with the flow voids might provide a clue to distinguish lacunae from lakes. On the other hand, the flow voids were not observed in two patients with PAS. Flow voids are a signal loss due to blood flow in spin echo sequences. The degree of signal loss is associated with the velocity of the proton out of the plane, the slice thickness, and the echo time. Therefore, the velocity and the imaging plane might contribute to the lack of flow void in diffusion lacunae. Additionally, the slice thickness was relatively large in some cases. Thus, the lack of signal loss was suggested to be due to the thick slice thickness. Meanwhile, DWI enables direct demonstration of the placental lacunae as hypointensity areas regardless of blood flow velocity or normal slice thickness. Based on these considerations, both the identification of diffusion lacunae and confirmation of T2 flow voids within the diffusion lacunae are important for evaluating PAS.

Our results also showed that all the standard MR imaging findings except for bladder wall interruption were also significantly correlated with PAS, which is consistent with previous reports. Placental bulge had the highest accuracy with high specificity, followed by our novel findings of diffusion lacunae. T2-dark bands, loss of T2 hypointense interface and abnormal vascularization of the placental bed also showed relatively high accuracy with high specificity; these were similar to the findings in previous reports [41–44]. Thus, our novel MR finding of diffusion lacunae may have diagnostic utility equivalent to that of the SAR-ESUR recommendations. Moreover, the diagnostic performance might be improved by combining the assessment of standard MR findings with the findings of diffusion lacunae, although further research with a larger number of cases will be needed to confirm this. Meanwhile, bladder wall interruption was observed in only 2 cases in the PAS group, resulting in low sensitivity and accuracy. Although bladder wall interruption is very useful findings for the diagnosis of percreta, there are few opportunities to contribute to the diagnosis of PAS due to its low occurrence rates [23, 45, 46].

Our study has several limitations. First, the sample size was relatively small due to the rarity of PAS. For the same reason, the analysis based on the depth of placental invasion and multivariate analysis on each MR finding were also difficult. The scoring using the combination of the diffusion lacunae and the flow void might predict the depth of invasion, similar to US lacunae grade [11]. Second, the slice thickness of MR images varied among the cases, which may have influenced the evaluation of the MR imaging findings. Third, because this was a retrospective study, we were not able to compare the location of diffusion lacunae on MRI with that of placental lacunae on US.

In conclusion, we reported novel MR imaging findings of PAS—namely, the placental lacunae on DWI and T2 flow voids within the diffusion lacunae. Diffusion lacunae and flow void within the lacunae may be useful findings for diagnosing PAS.
